# The Evolution of Biomineralization through the Co-Option of Organic Scaffold Forming Networks

**DOI:** 10.3390/cells11040595

**Published:** 2022-02-09

**Authors:** Smadar Ben-Tabou de-Leon

**Affiliations:** Department of Marine Biology, Leon H. Charney School of Marine Sciences, University of Haifa, Haifa 31905, Israel; sben-tab@univ.haifa.ac.il

**Keywords:** gene regulatory networks, evolution, biomineralization, tubulogenesis, skeletogenesis, vascularization

## Abstract

Biomineralization is the process in which organisms use minerals to generate hard structures like teeth, skeletons and shells. Biomineralization is proposed to have evolved independently in different phyla through the co-option of pre-existing developmental programs. Comparing the gene regulatory networks (GRNs) that drive biomineralization in different species could illuminate the molecular evolution of biomineralization. Skeletogenesis in the sea urchin embryo was extensively studied and the underlying GRN shows high conservation within echinoderms, larval and adult skeletogenesis. The organic scaffold in which the calcite skeletal elements form in echinoderms is a tubular compartment generated by the syncytial skeletogenic cells. This is strictly different than the organic cartilaginous scaffold that vertebrates mineralize with hydroxyapatite to make their bones. Here I compare the GRNs that drive biomineralization and tubulogenesis in echinoderms and in vertebrates. The GRN that drives skeletogenesis in the sea urchin embryo shows little similarity to the GRN that drives bone formation and high resemblance to the GRN that drives vertebrates’ vascular tubulogenesis. On the other hand, vertebrates’ bone-GRNs show high similarity to the GRNs that operate in the cells that generate the cartilage-like tissues of basal chordate and invertebrates that do not produce mineralized tissue. These comparisons suggest that biomineralization in deuterostomes evolved through the phylum specific co-option of GRNs that control distinct organic scaffolds to mineralization.

## 1. Introduction

The evolution of diverse life forms is one of the most complex natural phenomena that had fascinated scientists from many fields utilizing different approaches [1,2,3,4,5,6,7]. Molecular and genomic studies in the last decades revealed that instructions for the body plan are encoded in the genome in form of **gene regulatory networks (GRNs)**, with the initial conditions dictated by molecular and cellular information present in the egg [1,2,3,4,6,7,8,9,10,11,12,13]. Recent studies begin to illuminate how the genomic instructions are translated into cell specification and morphogenesis during embryogenesis [12,13,14,15,16], but deciphering how these genomic instructions evolve is still a major challenge.

A compelling approach to unravel the evolution of novel body plans is to compare the GRNs for cell fate specification and morphogenesis between different species [16,17,18,19]. These GRNs are not simple, but at least the regulatory interactions are well defined: transcription factors activate or repress the expression of other genes, including genes that encode transcription factors, signaling molecules and regulatory RNAs [20]. The feedback and feedforward regulatory circuitries define the set of transcription factors that is present in a cell nucleus and this set defines the gene expression profile within the cell, including the expression of differentiation genes [21]. **Differentiation genes**, in this context and throughout the manuscript, are genes that are expressed in a specific cell type, are important to its function and are not regulatory genes. In principle, if we want to understand how cell types evolved, we can compare the GRNs that drive them and track evolutionary progression.

However, the reality is more complicated, as GRNs can change quite rapidly within the phylum and even more so between different phyla [16,17,18,19,22]. This makes it challenging to detect the evolutionary trajectory of cell specific GRNs and conclude whether the diversification of the GRNs occurred before or after the diversification of phyla. Having said that, GRN comparison is a good starting point, and with the required caution, can reveal surprising evolutionary links that would otherwise be difficult to infer [17,22]. Here I focus on the GRNs that drive biomineralization in deuterostomes and compare relevant GRNs in echinoderms and vertebrates. This comparison portrays a possible scenario for the evolution of biomineralization by the co-option of ancestral GRNs that drive the formation of the organic scaffold wherein mineralization occurs.

## 2. Biomineralization and Its Evolution

**Figure 1:**
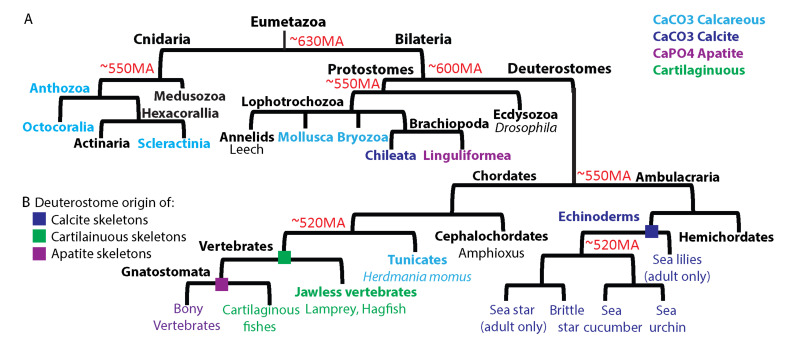
Partial Eumetazoan phylogenetic tree containing phyla and species discussed in this paper and their biomineralization programs. (**A**), calcification in cnidarian and protostome phyla. Numbers in red is the estimated time of divergence in million years (MA) based on [27,29]. Light blue indicates the usage of different CaCO3 polymorphs including but not limited to calcite, aragonite or vaterite. Dark blue indicates the usage of calcite, and purple indicates the usage of apatite. Black indicates non-mineralizing species. (**B**), skeleton formation in deuterostomes. Color code as in (**A**), with the addition of green, indicating the usage of cartilage by the skeletogenic tissue. Squares indicate the evolutionary origin of a specific skeletal tissue.

Biomineralization is the process in which organisms use minerals to generate hard structures like teeth, skeletons and shells that protect and support them [23,24,25]. The first animal skeletons are found in the late Ediacaran and early Cambrian periods and are related to the immense diversification of body plans at this time [7,26,27]. Except the siliceous skeletons found in sponges of the basal Porifera phylum [28], the skeletons of the rest of Metazoans, i.e., the Eumetazoa, are calcareous, made of calcium carbonate (CaCO_3_) or apatite (calcium phosphate, CaPO_4_, Figure 1A, [7,26,27]). Eumetazoa includes the basal phylum, Cnidaria and the clade, Bilateria and within both Cnidaria and Bilateria there are classes that produce calcified skeletons and classes that do not (Figure 1A). For example, the Cnidaria clade, Medusozoa, includes only soft-body organisms such as Jellyfish and Hydra; while the class Anthozoa contains subclasses such as Hexacorallia that includes both soft-body orders like the Actiniaria (sea anemones) and calcifying orders such as Scleractinia (stony corals, Figure 1A). The mineral used by Cnidaria is calcium carbonate in its various polymorphs of calcite, aragonite and vaterite [7,26,27]. Bilaterians use calcium carbonate and apatite, sometimes in the same clade, e.g., the Brachiopoda consists of the Chileata class that generates calcite shells and the Linguliformea subphylum that includes mostly apatite forming species (Figure 1A, [27]). Thus, within Eumetazoa, biomineralization was rapidly gained using various calcium related minerals and polymorphs.

Within the diverse mineral usage and biomineral shapes, in all studied biomineralizing organisms, the mineral is secreted into an organic scaffold, sometimes called “the biomineralization compartment” [25,27]. The organic scaffolds show a significant diversity between different phyla, but within the phylum they are largely conserved (see in [26,27] and examples below). Furthermore, the organic scaffold can be shared between mineralizing and non-mineralizing organisms within the same clade, as in vertebrates (Figure 1B). The bony vertebrates generate apatite skeletons by the mineralization of a bone matrix mold that replaces a cartilage mold made by chondrocytes [30,31,32]. The cartilage scaffold makes the skeleton of the non-calcifying cartilaginous fishes and jawless vertebrates (Figure 1B, [33,34]). The cartilage scaffold is probably the ancestral structure adapted for biomineralization in the bony vertebrates by the evolution of calcifying cells [33,34]. Thus, the organic scaffold makes the mold in which biominerals are formed and is apparently inherited from a non-biomineralizing ancestor.

The rapid acquisition of biomineralization in different phyla is proposed to have evolved independently through the co-option of pre-existing developmental programs and the evolution of specialized biomineralization proteins [7,26,27]. Co-option is the redeployment of pre-existing molecular traits for a new function, and is believed to be a major driver of the evolution of novel traits [35,36]. One of the most studied examples for protein co-option is the re-use of heat-shock proteins as the crystallins that make the vertebrates’ lens [35]. Here the proteins are used for a completely novel function, possibly due to their optical and stress resisting properties. There are also instances of a co-option of entire regulatory programs, e.g., beetles use a gene regulatory circuit that regulates limb development of other insects to generate the new structure of the beetle horns [37]. Hence, GRN co-option is the activation of an ancestral GRN in a new embryonic location and modification of some of the GRN linkages and downstream targets, so the co-opted GRN drives the morphogenesis of a new organ.

**Figure 2:**
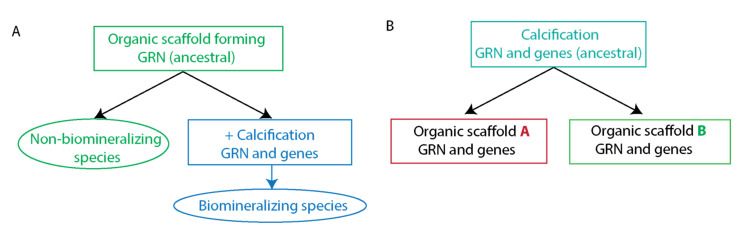
Possible models for the evolution of biomineralization GRNs from ancestral GRNs. (**A**), a model where an ancestral GRN that drove the formation of an organic scaffold was co-opted for biomineralization by activating regulatory and differentiation genes that assist in calcification. In this model we expect to see a strong similarity between the biomineralization of GRN to the GRN that drive the formation of the organic scaffold in non-biomineralization species. (**B**), a model where an ancestral GRN that drove the activation of genes that participate in calcification was co-opted for biomineralization through the activation of phylum specific regulatory and differentiation genes that control organic scaffold formation. In that case, we expect to see a similarity between the regulatory and differentiation genes that drive calcification in the two phyla.

Possibly, the GRNs that drive the formation of the organic scaffold were co-opted for biomineralization by the insertion of a GRN module that controls the expression of newly evolved specialized biomineralization genes (Figure 2A). In this case, we would expect to see similar regulatory circuits that control the organic scaffold formation in the biomineralizing and non-biomineralizing species that evolved from a common ancestor that had the organic scaffold. Alternatively, biomineralization could have evolved by the incorporation of an ancestral GRN that drives the expression of mineral binding and homeostasis proteins into a different scaffold generating GRNs (Figure 2B). In that case, we would expect to see common regulatory circuits that control biomineralization related processes in the two branches. These two models are not necessarily mutually exclusive, and the biomineralization GRN could be composed of calcification GRN modules and organic scaffold modules that are shared between the branches. Below I examine these possibilities by comparing the GRNs that drive the organic scaffolds and those that drive biomineralization in echinoderms and vertebrates.

## 3. Skeletogenesis in Echinoderms and the GRNs That Control It

The echinoderm phylum shares a close ancestry with the vertebrate phylum and provides an excellent platform for studying the structure and evolution of biomineralization GRNs [15,17,18,38,39,40,41]. All echinoderm classes generate calcite endoskeleton in their adult form [39,42,43]. Two echinoderm classes, the brittle stars (Ophiuroids) and sea urchin (Echinoids) develop a full larval skeleton early in embryogenesis, and a degenerate skeleton develops in the sea cucumbers (Holothuroids, Figure 1B) [39,42,43]. The GRNs that control skeletogenesis were studied in the both larval and adult skeletons in multiple echinoderm classes, which provides a unique opportunity for intra-phylum comparison. In this section, I first describe the biomineralization process in adult echinoderms and in the larva of the sea urchin, then I review the structure of the GRN that drives larval skeletogenesis in the sea urchin embryo. Finally, I discuss the conservation of this GRN within echinoderms while refereeing to relevant mesodermal GRNs.

### 3.1. Adult and Larval Skeletogenesis in Echinoderms

The adult echinoderm skeleton is made of porous calcite ossicles, arranged in plates and spines [44,45,46]. The ossicles are formed within multinucleated syncytia of sclerocytes placed in the dermal layer of the body wall [44,45]. The adult skeleton of the sea urchins was the focus of intensive research in the biomineralization field, which led to major discoveries regarding the crystallization pathway [47,48]. Nevertheless, the larval skeleton of the sea urchin embryo provides a more accessible model for studying GRN structure [15,41] and mineral growth [49,50,51].

**Figure 3:**
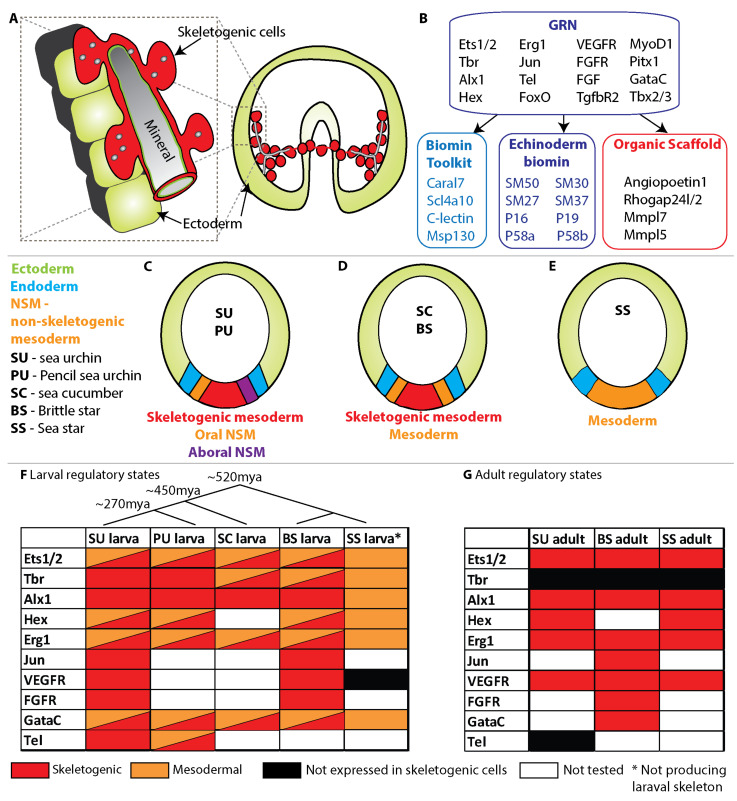
Sea urchin skeletogenic GRN and the evolution of echinoderm skeletogenic GRN. (**A**), a scheme showing larval skeleton formation in the sea urchin embryo. The skeletogenic cells (red) form a ring with two lateral skeletogenic clusters where the spicule form. Enlargement, showing the mineral (gray) concentrated in vesicles and transported to the spicule tubular compartment where it is engulfed within a thin layer of extracellular matrix. (**B**), sea urchin larval skeletogenic GRN and differentiation genes with various functions. (**C**–**E**), embryonic territories in the different echinoderm clades. Color codes are explained in the figure and in the text. C, embryonic territories in the sea urchin (SU) and the pencil sea urchin (PU). D, embryonic territories in the sea cucumber (SC) and brittle star (BS). (**E**), embryonic territories in the sea star (SS). (**F**), expression of the skeletogenic regulatory genes in the mesoderm of embryos of different echinoderm clades. Color code explained in figure. (**G**), expression of skeletogenic regulatory genes in adult skeletogenic cells in three echinoderm clades.

The larval skeleton of the sea urchin embryo consists of two calcite spicules generated by the skeletogenic lineage. Like the adult sclerocytes, the skeletogenic cells fuse to each other and form a syncytium (Figure 3A, [52,53]). The skeletogenic cells generate the spicules inside a tubular cavity that they form through the secretion of mineral bearing vesicles (Figure 3A, [50,51,54,55]). The thin organic matrix within the tubular cavity includes glycoproteins but no trace of collagen [56,57]. The collagen made by skeletogenic cells is secreted into the blastocoel [58], possibly to support their adhesion to the ectodermal cells (Figure 3A). Thus, the organic scaffold in this case is the tubular compartment that the skeletogenic cells form in which the calcite spicules grow.

### 3.2. The GRN and Differentiation Genes That Control Sea Urchin Larval Skeletogenesis

The GRN that drives skeletogenesis in the sea urchin embryos was extensively studied in the last two decades, resulting in one of the most comprehensive models of developmental GRN [15,40,59,60,61,62,63]. The GRN contains about 20 transcription factors that control a sequence of morphogenetic processes, including epithelial to mesenchymal transition, cell migration and cell fusion [15,40,64,65]. Some of the early transcription factors regulate these early morphogenesis events [64] but shut down before mineral deposition [15]. Here I focus on the GRN circuits that regulate biomineralization, therefore I include only the regulatory genes whose skeletogenic expression is maintained during mineral deposition (Figure 3B). Relatedly, since biomineralization and scaffold formation are driven by enzymes and other cellular proteins, I enlist key skeletogenic differentiation genes that participate in biomineral formation.

Despite the importance of the regulatory links between the genes to GRN function, I chose not to show these links since they change throughout development, and most of them were only studied during early skeletogenesis [15,18]. Furthermore, the regulatory links are less conserved within the phylum than the set of genes in the GRN, and even more so between phyla [17,66,67,68,69,70,71,72,73]. Therefore, throughout this manuscript, I list the regulatory and differentiation genes that are part of a GRN and not the links between them, in order to allow the comparison of the basic structure of the GRNs between diverse cell types and species.

A set of transcription factors including Ets1/2, Tbr, Alx1, Hex and Erg, is expressed in the skeletogenic cells from the early stages of skeletal specification and throughout skeletogenesis (Figure 3B, [15,18,74,75]). Perturbing the expression of each of these transcription factors results in significant skeletogenic phenotypes [15,59]. Together, they turn on downstream biomineralization genes as well as other regulatory genes, including the transcription factors Jun, FoxO and Tel as well as signaling genes, such as the Vascular endothelial growth factor Receptor (VEGFR) and Fibroblast Growth Factor (FGF) ligand and Receptor (FGFR) (Figure 3B, [15,75,76]). VEGF signaling is essential for spicule formation [61,75], and FGF signaling is important for skeletal elongation [61,76]. The transforming growth factor beta (TGFβ), whose receptor, TGFβR2, is expressed in the skeletogenic cells, is also important for skeletal elongation [77]. At the time of spicule formation, VEGF signaling activates the expression of the transcription factors, MyoD1 and Pitx1, in the lateral skeletogenic cell clusters [18]. Bone morphogenetic protein (BMP) signaling drives the expression of the transcription factors Tbx2/3 and GataC in dorsal skeletogenic cells [78]. Thus, a set of transcription factors and signaling pathways is expressed in the skeletogenic cells and controls skeletal formation and elongation through the activation of regulatory and differentiation genes.

The differentiation genes activated by the skeletogenic GRN regulate sea urchin biomineralization and, apparently, the construction of the organic tubular scaffold. Within these differentiation genes, we can find genes that participate in calcification (generating a calcium based mineral) in other organisms that are represented here with a few examples. The gene that encodes the carbonic anhydrase enzyme, Caral7, is activated by VEGF signaling in the skeletogenic cells (Figure 3B, [18]). Carbonic anhydrase catalyzes the reversible hydration of carbon dioxide and is a key biomineralization protein in metazoans that use calcium carbonate [79]. The gene that encodes the solute carrier bicarbonate transporter, Scl4a10, participates in pH regulation in the skeletogenic cells [80]. Homologs of this gene are expressed in the sclerocytes of calcareous corals and sponges, demonstrating its ancient role in calcium homeostasis [81,82]. Genes that encode c-lectin glycoproteins and MSP130 glycoproteins are expressed in the skeletogenic cells and are highly abundant in the larval and adult skeleton [83]. MSP130 is also expressed in mineralizing cells in invertebrates [84] and a c-lectin ortholog is expressed in mice osteoblasts and contributes to the maintenance of mice adult skeletons [85]. Caral7, Scl4a10, C-lectin and Msp130 could be a part of a conserved biomineralization tool-kit that existed in the ancestral metazoan and adapted by echinoderms and other phyla for calcification.

In addition to the shared biomineralization genes, there are echinoderm specific biomineralization genes expressed in the skeletogenic cells [86,87]. The proteins encoded by these genes are found in the larval and adult skeletons of sea urchins and other echinoderms [66,67,83,86,88,89,90]. These are the spicule matrix proteins, SM50, SM30, SM27 and SM29, as well as other phylum specific proteins such as p16, p19, p58a and p58b [41,66,67,83,86,90,91,92]. These genes are activated by the skeletogenic GRN [15,41], participate in biomineralization process [91,92,93,94] and must have evolved specifically in the echinoderm phylum to regulate mineral phase and shape [95].

Some of the skeletogenic differentiation genes have homologs involved in vascular tubulogenesis in vertebrates, and could be a part of the molecular mechanism that build the tubular spicule scaffold. Sea urchin VEGF signaling drives the expression of the signaling molecule, Angiopoetin1 in the skeletogenic cells [18], and the vertebrate homolog of Angiopoetin1 is essential for vascular maturation [96]. The cytoskeleton remodeling gene, *rhogap24l/2*, is activated by sea urchin VEGF signaling at the skeletogenic lateral cell clusters just before spicule formation and its expression is necessary for normal skeletal branching [18]. A vertebrates’ homolog of this gene is enriched in endothelial cells and is essential for vascular tube formation [97]. The membrane type metalloproteinases, Mmpl5 and Mmpl7, are activated by VEGF signaling at the skeletal growth zone at the tips of the rods, and Mmpl7 is important for normal spicule elongation [63]. The proteolytic activity of a vertebrates’ homolog of these genes, MT1-MMP, is essential for vascular tubulogenesis [98,99]. Thus, genes that encode proteins that participate in vertebrate vascular tubulogenesis are activated by the skeletogenic GRN and apparently control different aspects of the formation of the spicule tubular compartment.

### 3.3. Echinoderm Skeletogenic and Mesodermal GRNs

To understand the evolutionary origin of the sea urchin larval skeletogenic GRN, it is helpful to put it in the context of the larval mesodermal GRNs and the adult skeletogenic GRNs in the sea urchin and other echinoderms. The non-skeletogenic mesoderm (NSM) in the sea urchin gives rise to muscles, a coelomic pouch, blastocoelar cells and pigment cells [16,100,101]. The pigment cells originate from aboral (dorsal) NSM, have an immune function and are an evolutionary innovation of the echinoids (Figure 3C, [17,102,103]). The blastoceolar cells originate from the oral (ventral) NSM and are essentially hemocytes (blood cells) that are a part of the sea urchin immune system (Figure 3C, [104]). Blastoceolar cells are a part of the mesodermal lineage in all echinoderms, including the sea star, which lacks a larval skeleton and pigment cells (Figure 3D,E [72,73]). In this section, I discuss the mesodermal expression of sea urchin skeletogenic genes that were shown to be expressed in the mesodermal GRNs in at least one more species (Figure 3F).

Some of the skeletogenic regulatory genes are exclusively expressed in the sea urchin skeletogenic cells, while others are expressed also in other mesodermal territories (Figure 3F [102,105]). Explicitly, the transcription factors Tbr, Alx, Jun and Tel, and the signaling receptors VEGFR and FGFR are exclusively expressed in the skeletogenic cells while Ets1/2, Erg1, Hex and GataC are also expressed in the oral NSM cells (Figure 3C,F [59,75,102,105,106]). Relatedly, when the skeletogenic cells are removed from the sea urchin embryo, some of the oral NSM cells differentiate into skeletogenic cells and generate a skeleton [107,108,109]. This trans-differentiation occurs in the blastocoelar cells that express low levels of VEGFR which enable them to respond to VEGF signaling when the skeletogenic cells are removed and transform into skeletogenic cells [110]. Thus, in the sea urchin embryo, some regulatory genes are shared between the skeletogenic and blastocoelar GRNs and the blastocoelar cells can switch into skeletogenic fate through the activation of VEGF signaling.

The larval skeletogenic and mesodermal GRNs were studied in the pencil sea urchin [69,71,111], the sea cucumber [73] and the brittle star [68,90,112] and show resemblance to the sea urchin mesodermal expression patterns (Figure 3F). The pencil sea urchin diverged from the sea urchin about 270 million years ago, and its larval skeleton develops in later developmental stages relative to the sea urchin skeleton [69]. Yet, the pencil sea urchin and modern sea urchins have similar mesodermal lineages, show similar mesodermal expression patterns and their overall skeletal morphology is comparable (Figure 3C,F [69,111]). The sea cucumbers diverged from the echinoids about 450 million years ago [113] and develop a small degenerate skeleton in their larval stage [39,73]. The transcription factors Ets1/2, Tbr, Erg1 and GataC are expressed in both the skeletogenic and non-skeletogenic mesoderm, while Alx1 is exclusively expressed in the skeletogenic cells of the sea cucumber embryo (Figure 3D,F [73]). The brittle stars diverged from the echinoids about 520 million years ago and develop a full size larval skeleton [39,113]. Despite the long evolutionary distance, the skeletogenic and mesodermal gene expression are very similar between the brittle star and the sea urchin (Figure 3F). Importantly, Alx1, Jun, VEGFR and FGFR are skeletogenic specific, while Ets1/2, Hex, Erg1 and GataC are also expressed in the NSM [112]. These genes are also expressed in the skeletogenic cells of the adult sea urchin [114,115], brittle star [45,66,67,68] and sea star ([114,116], Figure 3G). The similarity of the skeletogenic regulatory states between the different species in the larval and adult stages suggests a strong conservation and a common origin of the core biomineralization GRN within echinoderms.

The skeletogenic regulatory states in the echinoderms that produce the larval skeleton are quite similar to the mesodermal regulatory state in the sea star that lacks this structure, which could explain the quick gain or loss of the larval skeletogenic program (Figure 3E,F). The sea star mesodermal cells express the transcription factors Ets1/2, Tbr, Hex, Erg1 and GataC, but do not express the signaling receptor VEGFR [17,72,116,117]. The transcription factor Alx1 was observed in the embryonic mesodermal cells of the sea star *Patiria Miniata* [73] but not in the species *Asterina pectinifera,* where it was only observed in mesenchymal cells at the bipinnaria larval stage [118]. Thus, it seems that the regulatory state in the sea star larval mesoderm was permissive for the activation of the skeletogenic program through just a few regulatory changes. Overall, the skeletogenic GRN shows high conservation within the echinoderm phylum, which makes it intriguing to compare it to the biomineralization GRNs in the relatively close vertebrate phylum.

## 4. GRNs That Drive Biomineralization in Vertebrates

### 4.1. Biomineralization Programs in Vertebrates

**Figure 4:**
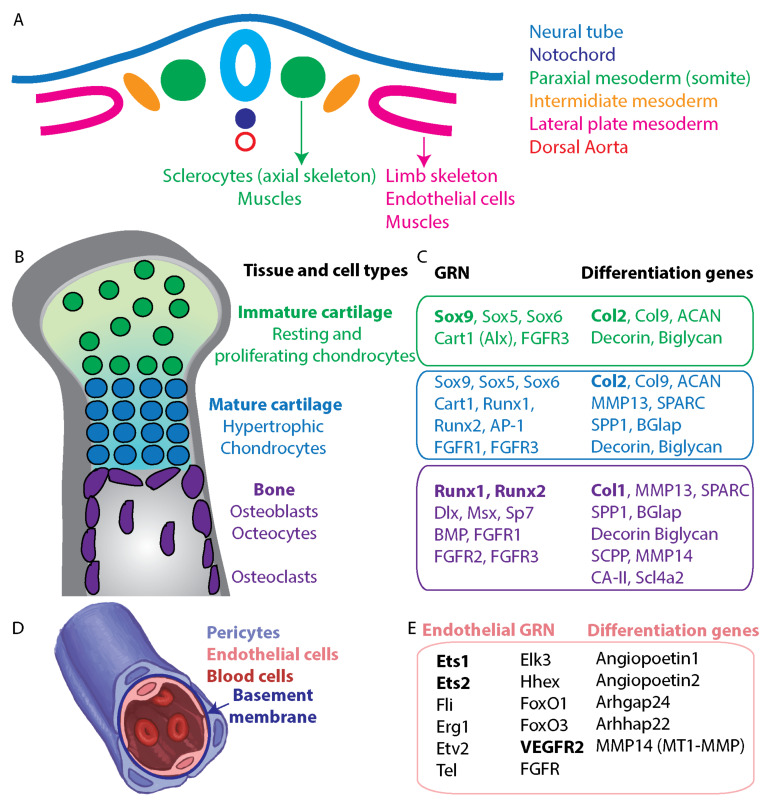
Bone biomineralization and vascular tubulogenesis GRNs in vertebrates. (**A**), schematic diagram showing a cross section of the dorsal part of a vertebrate embryo and the different embryonic territories that contribute to skeletal and vascular tissues. (**B**), tissues and cell types that participate in endochondral ossification in vertebrates. Immature cartilage (green) is generated by proliferating and resting chondrocytes. Mature cartilage (blue) is generated by hypertrophic chondrocytes, bone matrix and mineralization (gray) are generated by osteoblasts, maintained by osteocytes and reabsorbed by osteoclasts (purple). (**C**), GRN and differentiation genes in the different bone forming cells. Color code matches the territories and cells in (**B**,**D**), schematic diagram of vertebrate blood vessel showing the different cell types that constitute it. Blood cells occupy the lumen which is engulfed by endothelial cells. The endothelial cells are bound to the basement membrane from the inner side of the vessel and pericytes from the outside. Image courtesy of Yarden Ben-Tabou de-Leon (artist). (**E**), endothelial cell GRN and differentiation genes.

The vertebrate phylum is a relatively close phylum to echinoderms, and its biomineralization GRNs and their evolution were comprehensively studied (Figure 1, [31,33,119,120,121]). The vertebrates’ mineralized tissues are mainly bones and teeth, and they originate from the ectoderm (enamel), the neural crest (craniofacial bones and dentin), the paraxial mesoderm (vertebral and craniofacial bone) and the lateral plate mesoderm (limb bone, Figure 4A [31,122]). Bone formation involves the transformation of preexisting mesenchymal tissue into bone tissue. This transformation occurs either through direct deposition of bone matrix by **osteoblasts**, termed intramembranous ossification, or through the replacement of a cartilage template by a bone tissue termed endochondral ossification [122,123]. Since echinoderm skeletogenic cells are mesodermal, I focus here on the mesodermal derived biomineralization GRNs and, specifically, on the GRNs that drive endochondral ossification in vertebrates and their evolution.

### 4.2. Endochondral Ossification in Vertebrates

Endochondral ossification is characteristic for vertebrae, ribs and limb formation [122]. In this process, the bone forms through the sequential calcification of cartilage and bone matrix molds [122]. At the first stage, the mesenchymal cells that originate from the paraxial or lateral plate mesoderm are condensed and differentiated into proliferating chondrocytes (green cells in Figure 4B, [31,122]). The chondrocytes deposit an extracellular matrix, mainly, **collagenII (Col2)**, to make a cartilage mold [31]. The chondrocytes then organize into columnar structures and differentiate into post-mitotic hypertrophic chondrocytes (blue cells in Figure 4B, [31]). The hypertrophic chondrocytes secrete osteogenic factors, which leads to the differentiation of the osteoblasts from a thin layer of progenitor cells originating from the condensed mesenchyme at the margin of cartilage (purple cells in Figure 4B, [31]). Additionally, hypertrophic chondrocytes can transdifferentiate into osteoblasts, and this process contributes significantly to the osteoblasts population [124].

The osteoblasts replace the cartilage with bone matrix, mainly, **collagenI (Col1)**, and mineralize the bone matrix [125]. Osteoblasts that are buried in the bone differentiate into osteocytes and participate in bone remodeling in the mature bone [126,127]. Bone remodeling is the resorption of the mineralized bone matrix by **osteoclasts** and the formation of a new mineralized bone matrix by osteoblasts [31]. Thus, bone development and remodeling are driven by cartilage forming chondrocytes and hypertrophic chondrocytes, bone matrix forming and mineralizing osteoblasts, and bone resorbing osteocytes and osteoclasts.

### 4.3. GRNs That Drive Endochondral Ossification in Vertebrates

The transition between the secretion of Col2 in chondrocytes to Col1 in osteoblasts is regulated by a switch from a GRN dominated by Sox transcription factors to a GRN dominated by Runx’s (Figure 4C, [34,125,128]). In the proliferating chondrocytes the transcription factor, Sox9 is expressed in high levels together with Sox5, Sox6, Cart1 (homolog of Alx1 [129]) and the signaling receptor FGFR3 [34,121,130]. These regulatory genes drive the expression of Col2, as well as the expression of the cartilage proteoglycan, aggrecan (ACAN) and proteins from the small leucine-rich repeat proteoglycan (SLRP) family, Decorin and Biglycan [31]. During the differentiation of the proliferating chondrocytes into hypertrophic chondrocytes, the expression of the Sox transcription factors decreases and transcription factors Runx1 and Runx2 are activated (Figure 4C, [34,121,125]). Other regulatory genes that turn on during this transition encode the transcription factor AP-1 (Jun-Fos) and FGFR1 [34,121,125,131,132]. The activation of Runx transcription factors in the hypertrophic chondrocytes drives the expression of osteogenic factors such as the fibrillar collagen binding protein, SPARC, the acidic secreted phosphoprotein, SPP1, the bone Gla protein, BGlap and MMP13 that specifically degrades Col2 and ACAN (Figure 4C, [30,31,34]). Thus, the differentiation of hypertrophic chondrocytes is regulated by the downregulation of Sox and the upregulation of Runx transcription factors, which induce the activation of osteogenic factors and Col2 degrading enzymes.

The regulatory state of the osteoblasts is dominated by Runx1 and Runx2 and the transcription factors Dlx, Msx and Sp7, while the transcription factors Sox9, Sox5, Sox6 and Alx1 are turned off [34,125,133]. As mentioned above, many of the hypertrophic chondrocytes differentiation genes are also expressed in osteoblasts, yet there are some clear differences. First, osteoblasts express Col1 instead of Col2, and, accordingly, the Col2 binding protein, ACAN, is not expressed in osteoblasts (Figure 4C, [34]). The osteoblasts express other bone matrix proteins such as the secretory calcium-binding phosphoproteins (SCPP, [31]) and the membrane type metalloprotease, MMP14 (MT1-MMP). MMP14 regulates the apoptosis of chondrocytes and the remodeling of unmineralized cartilage necessary for postnatal skeletal remodeling [134,135]. Thus, the osteoblasts’ GRN drives the expression of proteins that drive chondrocytes apoptosis, degrade the cartilaginous matrix, and replace it with a bone matrix that they mineralize.

### 4.4. The Evolutionary Origin of the Endochondral Ossification GRNs

Chondrocytes, hypertrophic chondrocytes and osteoblasts originate from the same mesenchymal progenitors and their GRNs show a clear transition from cartilage forming to bone matrix forming, which could recapitulate the evolution of the bone [34,128,136]. Indeed, the bone GRNs were proposed to have evolved from an ancestral cartilage forming GRN that was co-opted for biomineralization in the vertebrate phylum [34,128,136]. Thus, we could imagine two clear processes in the evolution of the bone: First is the evolution of the vertebrate cartilage GRN and the second is its co-option for biomineralization. The evolution of cartilage and the evolution of vertebrate biomineralization were extensively reviewed by others [31,34,121,128,136,137,138,139], so here I briefly summarize the main points.

The cartilage GRN seem to have originated in the bilaterian ancestor [139] and evolved within the chordates [33,140,141]. The co-expression of SoxE (invertebrate ortholog of Sox8/9/10), SoxD (ortholog of Sox5/6) and ColA (ortholog of Col2) in cells that generate cartilage like structures were observed in two diverged protostomes, the horseshoe crab and the cuttlefish [136]. This dates the origin of cartilage GRN to the common bilaterian ancestor. Within the deuterostomes, co-expression of SoxE and Col2 genes was detected in endodermal derived cells near the collagenous cartilage skeletons that support cephalochordates and hemichordates pharyngeal gill slits [142,143]. The cartilaginous skeleton of Lamprey, a jawless vertebrate, contains Col2, and Sox9 is co-expressed with Col2 during skeletal development [140]. Thus, the cartilage forming GRN dominated by SoxE, SoxD and driving the expression of type II collagen, had probably originated in the bilaterian ancestor, evolved within the deuterostomes and co-opted for biomineralization in the vertebrate phylum.

The chordate cartilage GRN was co-opted for mineralization, apparently through the upregulation of the *runx* genes that acquired new targets, the downregulation of the *sox* genes and the evolution of a new set of bone matrix proteins [31,128]. The molecular mechanism enabling some of these evolutionary changes is apparently the two events of whole genome duplication that occurred during vertebrate evolution, and lead to the generation and specialization of new sets of orthologous genes [31,34]. For example, the single ancestral chordate *runx* gene duplicated into the three vertebrates *runx* genes [144,145,146] and the ancestral SoxE gene duplicated into the vertebrates’ sox8, sox9 and sox10 [34], possibly enabling a rewiring of their regulatory connections. Moreover, the duplication of the ancestral SPARC gene lead to the evolution of the SPARC-like gene and to its tandem duplication that lead to the evolution of the vertebrates’ SCPP family that is an essential component of the bone matrix [147]. Overall, the expansion and specialization of regulatory and biomineralization gene families in the vertebrate phylum supported the co-option of the chordate cartilage GRN to biomineralization.

### 4.5. Vertebrates’ Bone GRNs vs. the Echinoderm Skeletal GRNs

The vertebrate bone GRNs are quite distinct from the echinoderms core skeletogenic GRN (Figure 3F and Figure 4C). There are only a few regulatory genes common to both GRNs, namely, Alx1, AP-1 and FGFR and within these genes, Alx1 and AP-1 are expressed in the cartilage forming chondrocytes and turn off in the biomineralizing osteoblasts [125]. Furthermore, orthologs of the key regulators of the cartilage and bone GRNs, SoxE, SoxD and Runx, are not expressed in the sea urchin skeletogenic cells, but in mesodermally derived cells at the tip of the gut [16,106,146,148]. Sea urchin *runx* is also expressed in the dorsal ectoderm [146]. Importantly, while the vertebrate organic scaffold is collagenous and the biomineral that forms is apatite, collagen is not found in the sea urchin skeleton [56,57], many other matrix genes are phylum specific [31,83], and the biomineral that forms is calcite. Collagen is expressed in skeletogenic cells in both sea urchin larva [58,149] and adult brittle stars [45], possibly to strengthen the extracellular matrix around the biomineralized spicules. Yet, differently than in bone, the collagen secreted by the echinoderm skeletogenic cells is not mineralized. Thus, the clear differences in the usage of regulatory genes, matrix proteins and minerals between the vertebrate bone GRNs and the sea urchin skeletogenic GRN do not support a common origin of these two biomineralization GRNs.

## 5. GRNs That Drive Vascular Tubulogenesis in Vertebrates

Echinoderm biomineralization occurs within a tubular cavity formed by the skeletogenic cells, and the core GRN that controls this process, shows a strong similarity to the endothelial cell GRNs that control vascularization in vertebrates (Figure 3F and Figure 4E [18,65]). Despite the distinct structural and functional differences between sea urchin skeletons and vertebrates’ blood vessels, an ancestral GRN that controls tube formation could have evolved for these separate usages in the two phyla. Below I review the morphogenetic processes that drive blood vessel formation in vertebrate, the GRN that regulates these processes, the evolution of this GRN, and its resemblance to the echinoderm skeletogenic GRN.

### 5.1. Vertebrates’ Endothelial Cells, Vascularization and Angiogenesis

Vertebrates’ blood vessels form during embryonic development in adult ischemic tissues and in pathological conditions such as cancer [122,150,151,152]. The first blood vessel that forms during vertebrate development is the dorsal aorta generated by endothelial cells that originate from the lateral plate mesoderm (LPM, Figure 4A, [122,151,152,153]). Endothelial progenitor cells migrate from the LPM to the medial region and generate a chord that they fill with lumen to form the dorsal aorta [153,154,155]. The hematopoietic stem cells emerge from a subpopulation of endothelial cells located at the ventral wall of the dorsal aorta [153]. The vascular network then expands through the process of sprouting angiogenesis, the formation of new blood vessels from existing ones [150].

Sprouting angiogenesis occurs through endothelial cell proliferation and directed migration toward the angiogenetic cues, followed by tubulogenesis, where the lumen extends into the new sprouts [150,156,157]. Lumen formation and extension in both embryonic vascularization and angiogenesis is driven by various molecular mechanisms including cytoskeletal remodeling, junctional rearrangement and vesicular transport [156,158,159,160,161]. Blood vessels maturation involves the formation of a basement membrane and the recruitment of pericytes and smooth muscle cells that coat the endothelial tube (Figure 4D, [162]).

### 5.2. The Endothelial GRN That Drives Vascularization and Angiogenesis

The GRNs that control embryonic vascularization and those that control angiogenesis were expansively studied in various vertebrate model systems during embryogenesis and in endothelial cell cultures [151,153,163,164]. In Figure 4E, I list key transcription factors, signaling pathways and differentiation genes that were shown to be expressed in endothelial cells and play an essential role in vascularization in multiple vertebrates’ models.

VEGF signaling and transcription factors from the ETS family are key regulators of endothelial specification and blood vessel formation. VEGF signaling is necessary for the migration of the angioblasts from the LPM, is essential endothelial cell specification, and critical for vascular tubulogenesis and for the induction of sprouting angiogenesis in healthy tissues and in cancer [150,153,165]. VEGF signaling induces the transcriptional activation of endothelial genes, partially through the acetylation of the ETS transcription factor [166]. Studies in zebrafish, *Xenopus*, mice and human endothelial cell cultures show that transcription factors from the ETS family including, ETS1, ETS2, Fli, Erg1, Etv2, Tel and Elk3, are key regulators of vascularization and angiogenesis and play various roles in endothelial cell differentiation, migration, tubulogenesis and vessel maturation [151,164,167,168,169,170,171,172,173,174,175,176]. Thus, the essential role of VEGF signaling and ETS transcription factors in endothelial cell specification and blood vessel formation in vertebrates is conserved within the phylum.

The regulation of angiogenesis requires negative cues provided by the transcription factors Hex and FoxO, and vessel remodeling and maturation cues by the signaling factors FGFR and angiopoietin (Figure 4E). The hematopoietically expressed homeobox (Hhex) is transiently expressed in endothelial cells during vascular formation and was shown to be a negative regulator of angiogenesis [177]. The forkhead transcription factors FoxO1 and FoxO3 are expressed in mature endothelial cells and negatively regulate postnatal vessel formation and maturation [178,179]. FGFR1 and FGFR2 are expressed in mature endothelial cells and regulate neovascularization and vascular remodeling after injury [180,181]. Angiopoietins are signaling ligands that bind to the receptor tyrosine kinase, Tie2, which is expressed in endothelial cells and regulates vascular maturation during developmental, physiological and pathological angiogenesis [96,182]. Angiopoietin2 is expressed in endothelial cells downstream of the transcription factor Ets1 [183]. Thus, Hex, FoxO, FGFR and angiopoietin are expressed in endothelial cells and regulate vascular homeostasis, vascular remodeling, and vessel maturation.

Blood vessel growth and lumen formation require the activity of cytoskeleton remodeling and extracellular matrix remodeling proteins activated by the endothelial GRN [156,184]. Among those, the Rho-GTPase activating proteins, Rhogap24 and Rhogap22, are among the most enriched RhoGAPs in endothelial cells (Figure 4E, [97,185].). Rhogap24 regulates endothelial cell migration, tubulogenesis and angiogenesis through the inactivation of the small GTPases, RAC1 and CDC42 [97,185]. The interactions between CDC42 and the membrane type Metalloproteinase MMP14 (MT1-MMP) are essential for vascular tubulogenesis and lumen formation [98,99]. Overall, lumen formation and tubulogenesis require the activity of the cytoskeleton remodeling proteins, Rhogap24 and CDC42 and the proteolytic activity of MMP14.

### 5.3. The Evolution of the Vertebrates’ Vascularization GRN

The endothelial cells that drive vascularization and form the inner lining of the vertebrates’ blood vessels, between the basement membrane and the lumen, are vertebrate specific [186,187]. In invertebrates’ vascular systems there are no true endothelial cells lining the lumen and, instead, the lumen is delineated by the basement membrane of surrounding epithelial cells. Thus, the endothelial cells are a vertebrate innovation, but blood vascular systems are common in invertebrates and comparative gene expression can illuminate the possible origin of the vertebrate vascularization GRN.

Within chordates, VEGFR expression and activity were linked to the development of the vascular system. The endothelial GRN that drives vascularization is highly conserved in vertebrates and especially the role of VEGF signaling and the ETS transcription factors [151,164,167,168,169,170,171,172,173,174,175,176,186,188]. Tunicates do not have true endothelial cells and their blood vessels contain lumen engulfed by basement membrane produced by the surrounding epithelial cells [189]. An ortholog of VEGFR is expressed in the epithelial cells that surround the peripheral blood vessel of the colonial tunicate, *Botryllus schlosseri,* and the inhibition of VEGFR signaling prevents blood vessel regeneration [189]. VEGFR homolog is enriched in tissues that contain blood vessels in the tunicate, *Halocynthia roretzi* [190]. In the cephalochordate Amphioxus, *Branchiostoma lanceolatum,* VEGFR ortholog is expressed in cells within the dorsal aorta and the subintestinal vessel and VEGFR inhibition reduces the level of Laminin in the basement membrane of the vessel [191]. These studies in basal chordates support the conserved role of VEGF signaling in blood vessel formation and of VEGFR expression in vascular epithelial cells.

Within the protostomes, VEGF signaling is necessary for the migration of blood cells in *Drosophila* and controls blood vessel formation in lophotrocozoan species [192,193,194]. In the arthropod, *Drosophila*, the ortholog of VEGFR is expressed in hemocytes [195] and three orthologs of VEGF are expressed along the hemocyte migration path [194]. *Drosophila* VEGF signaling is critical for hemocyte migration, proliferation and survival [194,196,197]. In *Hirudo medicinalis,* a leech from the annelid phylum, the injection of human VEGF induced the growth of new blood vessels [193]. This species has two VEGFR ortholog genes and immunostaining with the human VEGFR antibody showed expression in the walls of the blood vessels [193]. In *Idiosepius paradoxus* embryo, a squid from the mollusk phylum, an ortholog of VEGFR, is expressed in developing blood vessels and in the branchial arch [192]. Moreover, an ortholog of the transcription factor ETS is expressed in hemocytes and blood vessels in the mollusk, *Chlamys farreri,* a sea scallop [198]. The abovementioned studies show that VEGF signaling and, possibly, ETS factors are involved in hemocytes and blood vessel development in various protostome species.

Overall, the key role of VEGF signaling in vertebrates’ vascularization and hematopoiesis GRNs and VEGFs role in similar processes in other bilaterians imply that VEGF signaling was a part of an ancestral GRN that drove blood vessel morphogenesis. The expression of other genes in this GRN was less studied in invertebrates. Nevertheless, the strong regulatory links between the VEGF pathway and ETS factors in vertebrates and ETS expression in blood vessels and hemocytes of mollusk [198] suggest that ETS factors had been a part of the ancestral vascular GRN.

### 5.4. The Vascularization GRN vs. the Echinoderms’ Skeletal GRN

There is a remarkable similarity between the vertebrates’ endothelial GRN and the echinoderm skeletogenic GRN (Figure 4E and Figure 3F). These GRNs drive the formation of blood vessels and spicules, two tubular organs with distinct morphological structure and function: the endothelial cells are wrapped around the lumen and have cell-cell junctions between them [199], unlike the syncytial mesenchymal skeletogenic cells that are round, fused to each other and do not have junctions between them. Yet, the echinoderm skeletogenic cells form a tubular cavity that constitutes the organic scaffold in which their calcite spicule rods grow. Hence, both the endothelial GRN and the echinoderm skeletogenic GRN drive a process of tubulogenesis. Additionally, both GRNs are very close to hemocyte generating GRNs: The echinoderm skeletogenic GRN is highly similar to the NSM GRN that drives hemocytes specification and the hemocytes can re-differentiate into skeletogenic cells, depending on VEGF signaling (Figure 3D [110]). As explained above, a close relationship between hematopoeitic cells and endothelial cells as well as the ability to trans-differentiate between these fates exists in vertebrates [200]. Thus, the similarity in the GRN structure, tubular morphology and kinship to the hemocyte differentiation GRN support a common ancestral origin of the vertebrates’ vascularization and sea urchin skeletogenesis GRNs. Apparently, the common ancestral GRN drove vascularization and was uniquely co-opted for biomineralization in the echinoderm phylum.

## 6. Conclusions-Biomineralization Gene Regulatory Networks Evolve through the Co-Option of Organic Scaffold Forming Networks

**Figure 5:**
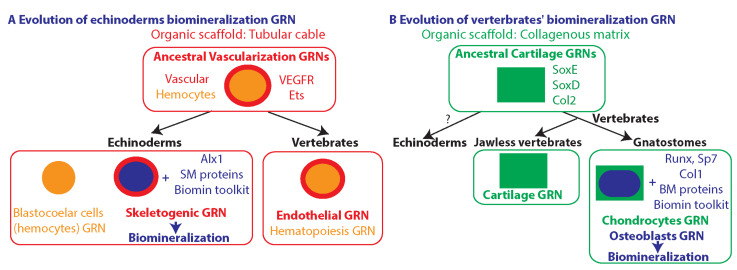
Proposed models of the evolution of the biomineralization GRNs in echinoderms and in vertebrates. (**A**), an ancestral vascular GRN that generated blood vessels where hemocytes flow evolved in vertebrates to make the endothelial GRN and was co-opted for biomineralization in echinoderms. The ancestral vascular GRN included the VEGF pathway and transcription factors of the ETS family. Co-option for biomineralization included the activation of the transcription factor Alx1, the evolution of novel echinoderm spicule matrix (SM) proteins and the activation of genes of the biomineralization toolkit. The vertebrate endothelial GRN and the echinoderm skeletogenic GRN show similarities and trans-differentiation potential to the hemocyte GRN. (**B**), an ancestral GRN that drove cartilage formation was co-opted for biomineralization in the vertebrate phylum. The ancestral cartilage GRN included the transcription factors SoxE and SoxD that drove the expression of Col2. The co-option for biomineralization was through the activation of the transcription factors Runx and Sp7, the evolution of novel bone matrix (BM) proteins and the activation of Col1 and genes of the biomineralization toolkit.

In this review, I examined two examples for the independent evolution of phylum specific biomineralization GRNs, the GRNs that control echinoderm skeletogenesis and the GRN that drives bone formation in vertebrates (Figure 3 and Figure 4B,C). The organic scaffold that echinoderms calcify is a tubular syncytial cable while the organic scaffold calcified by vertebrates is a collagenous extracellular matrix. The echinoderm core skeletogenic GRN shows little similarity to the vertebrate GRNs that drive biomineralization and strong resemblance to the GRN that drives vascular tubulogenesis (Figure 3F and Figure 4C,E). The vertebrates’ bone GRNs show similarities to ancestral GRNs that generate cartilage in bilaterians. This implies that these two phyla co-opted two distinct scaffold forming GRNs for biomineralization–echinoderms co-opted a vascular tubulogenesis GRN and vertebrates co-opted a cartilage generating GRN (Figure 5).

The echinoderms had apparently co-opted for biomineralization an ancestral vascularization GRN that included VEGF signaling and ETS transcription factors (Figure 5A, see references in Section 4). This co-option involved the activation of the transcription factor Alx1, the evolution of a novel set of genes encoding spicule matrix proteins and the activation of differentiation genes of the biomineralization toolkit, such as Caral7 and MSP130. Interestingly, Alx1 contains an echinoderm specific domain that is critical for its function in skeletogenesis [201]. The insertion of this region to the *alx1* gene could have been one of the genetic changes that contributed to the evolution of the echinoderms biomineralization program [201]. The endothelial GRN had probably evolved from the ancestral vascular GRN, utilizing the two events of whole genome duplication to generate the elaborate and highly specialized vertebrates’ vascularization programs. Both the skeletogenic GRN in echinoderms and the endothelial GRN in vertebrates show similarities and trans-differentiation potential to the hemocyte GRNs in the two phyla, possibly due to the close proximity between the ancestral hemocyte and vascular GRNs.

The bone GRN of vertebrates seems to have evolved from an ancestral cartilage forming GRN that contained the transcription factors SoxE and SoxD and drove the expression of Col2 and Col2 binding proteins (Figure 5B, [31,34,121,128,136,137,138,139]). The similarity of the cartilage GRNs between chordates and protostomes suggests that the main evolutionary innovations have occurred during the evolution of the bony vertebrates (Figure 5B). These innovations had apparently involved the activation of Runx and Sp7, the downregulation of SoxE and SoxD orthologs, a switch between Col2 to Col1, the evolution of a new set of bone matrix proteins and the activation of differentiation genes of the biomineralization toolkit, such as carbonic anhydrase II (CA-II) and the bicarbonate anion transporter Slc4a2 (Figure 4E and Figure 5B, [202,203]).

The two examples of biomineralization GRNs discussed here support the model proposed in Figure 2A for the evolution of biomineralization GRNs from an ancestral scaffold forming GRN. That is, the vertebrates’ bone GRNs and the echinoderm skeletogenic GRN show little resemblance between them and strong similarity to the GRNs that generate the distinct scaffolds that each GRN calcifies. However, there is a shared set of differentiation genes that is activated by both biomineralization GRNs, such as carbonic anhydrase, Slc4, etc. (Figure 3A and Figure 4E). These genes must have evolved in the early metazoan and are a part of a conserved biomineralization toolkit whose activation was acquired by the biomineralization GRNs of echinoderms and vertebrates, most likely independently. Apparently, the biomineralization GRNs in echinoderms and vertebrates evolved through the co-option of distinct organic scaffold GRNs and the activation of phylum specific as well as conserved biomineralization proteins (Figure 5).

There are still many open questions with regard to the evolution of biomineralization GRNs. The genomic mechanisms that allow for the tandem duplication and evolution of multiple calcium binding proteins that are phylum specific are yet to be identified. The activation of these newly evolved genes as well as the conserved biomineralization toolkit genes by the biomineralization GRNs possibly requires the parallel evolution of novel *cis*-regulatory elements of which we know very little. Finally, why a certain scaffold is specifically chosen for mineralization in a phylum is a question that will hopefully be answered by further research on the evolution of biomineralization GRNs and its relationship with the physiology of the species and with past environmental conditions.

## Data Availability

Not applicable.
